# Could Individual Variability in Resistance to Cryopreservation (“Freezability”) Serve as a Biomarker Reflecting Boar Fertility?

**DOI:** 10.3390/ani15152180

**Published:** 2025-07-24

**Authors:** Eduardo de Mercado, Helena Nieto-Cristóbal, Adrián Martín-San Juan, María José Martinez-Alborcia, Manuel Álvarez-Rodríguez

**Affiliations:** 1 Department of Animal Reproduction, Instituto Nacional de Investigación Agraria y Alimentaria-CSIC, 28040 Madrid, Spain; helena.nieto@inia.csic.es (H.N.-C.); adrian.martin@inia.csic.es (A.M.-S.J.); 2 Topigs Norsvin España SLU-AIM Ibérica, 28290 Las Rozas, Spain; mariajose.alborcia@aimiberica.com

**Keywords:** boar sperm, sperm quality, fertility, freezability

## Abstract

Subfertile boars often go undetected until they cause significant reproductive losses. Current semen quality assessments are limited in their ability to predict fertility, highlighting the need for complementary biomarkers. This study explored whether semen freezability could serve as an indirect indicator of boar fertility. Eighteen boars were classified based on historical fertility records and semen freezability, assessed by post-thaw quality. Fresh and post-thaw semen samples were analyzed using the CASA system and fluorescence microscopy. High-fertility boars showed significantly better motility and functional sperm parameters in fresh semen compared to low-fertility boars. However, these differences were mostly lost after cryopreservation. Conversely, boars with good freezability had consistently better post-thaw semen quality, though this did not correlate directly with higher fertility outcomes. Notably, a combined analysis revealed that boars with both high fertility and poor freezability had the lowest post-thaw semen quality. This suggests that cryopreservation may expose hidden sperm defects not detectable in fresh semen. Total motility was the only parameter associated with both fertility and freezability. In conclusion, while freezability alone may not directly predict fertility, it may help identify low-performing males. The combined assessment of fresh semen motility and freezability could support more effective boar selection strategies.

## Simple Summary

Detecting male pigs with low fertility early is a major challenge for pig farmers, as these animals often go unnoticed until they cause serious economic losses. In this study, we explored whether freezing semen could help reveal hidden fertility problems. We evaluated semen from several boars, analyzing its quality before and after freezing, and compared this with the fertility records of each animal. We found that boars with higher fertility generally had better semen quality before freezing, especially in terms of sperm movement. However, these differences mostly disappeared after freezing. On the other hand, boars whose semen handled freezing well did not always have higher fertility. Interestingly, semen from boars with both high fertility and poor resistance to freezing showed the lowest quality after thawing. This suggests that freezing might help uncover sperm problems that normal tests miss. While freezing ability alone does not predict fertility, combining it with sperm movement in fresh semen could help identify low-performing boars early. This would save time and money for the pig industry and improve overall breeding success.

## 1. Introduction

One of the major challenges in modern pig production systems is the presence of subfertile boars. Despite the high level of technological advancement, 5% to 10% of boars still exhibit below-average fertility [1,2]. When evaluating these using conventional semen quality parameters, such as viability and motility, these animals present optimal values, comparable to boars with normal fertility [3]. The difficulty in identifying subfertile boars at early stages means that these animals are incorporated into production systems for long periods of time, generating reproductive and economic losses [2]. These losses can be even more serious for farms when artificial insemination (AI) doses of semen from pooled ejaculates are used, as the poor fertility of subfertile boars may be masked by the higher fertility of others in the mix [2]. Therefore, the early identification of low-fertility boars will reduce the costs of breeding these animals and prevent delayed culling after suboptimal reproductive performance is observed [4,5].

Conventional semen analysis techniques have long been used to identify boars that may be at risk of reduced fertility due to suboptimal sperm quality. Among traditional parameters, correlations with decreased fertility have been reported for acrosome integrity [6], normal head and tail morphology [7,8,9], and motility [10]. Even parameters determined using more complex analyses, such as DNA integrity [10,11], as well as analyses based on fluorescence microscopy [12] or flow cytometry [9], have shown associations. Nevertheless, none of these parameters have been proven to be entirely reliable for identifying subfertile individuals. Indeed, some studies have found no significant associations between these markers and boar fertility [11,13].

Undoubtedly, the prior analysis of each ejaculate can provide us with real information about the future fertility of that ejaculate, particularly when considering the conventional semen quality parameters described above. However, the underlying problem arises when a boar with no prior abnormalities and normal semen parameters later develops reduced fertility. For this reason, a key goal in the swine industry is to search for new tools for the early detection of subfertile boars, not only through the better implementation of routine semen quality analyses but also by exploring other types of evaluations. These include potential differences in seminal plasma composition [14], the presence of specific proteins, enzymes, or miRNAs [1], and even resistance tests that may help correlate semen quality with fertility [15].

In recent years, significant progress has been made in the application of various -omics techniques (proteomics, genomics, epigenomics, transcriptomics, metabolomics, etc.), aiming to identify new biomarkers that predict boar fertility. Although promising findings have emerged, only a limited number of studies have applied these -omics tool approaches to directly compare fertile and subfertile boars [1,16,17,18,19].

For this reason, and because we are still trying to further understand the function of sperm, we must understand the needs of a sperm to achieve fertilization. A functional sperm must be motile, resilient enough to survive in the female reproductive tract, able to bind to and penetrate the zona pellucida, fuse with the oolemma, activate the oocyte, provide intact DNA, and trigger zygote development. The sperm’s ability to carry out all these processes depends largely on the state of its plasma membrane. This membrane maintains intracellular homeostasis and regulates the exchange of substances, energy, and information with the external environment. Membrane fluidity and permeability are critical determinants of sperm function [20,21,22]. The class and content of lipids present in the membrane directly determine these physicochemical properties, decisively influencing the viability and fertilizing capacity of the sperm [23,24,25].

A clear illustration of the critical role of the plasma membrane lies in its influence on a sperm cell’s resistance to cryopreservation. Freezability seems to have an individual character, and it has been shown that this may be due to the prevalence of certain lipids in the sperm plasma membrane in some boars compared to others [26,27]. In fact, recent findings have identified that levels of lipid metabolites, such as long-chain PUFAs, are key contributors to cryotolerance [25].

Moreover, some authors have even suggested that it is possible to predict the future fertility of a frozen ejaculate [28], further supporting the hypothesis of a link between sperm freezability and the fertilizing capacity of boar semen.

Therefore, the aim of this study was to investigate whether the resistance of boar sperm to cryopreservation (freezability) is associated with the fertility levels of the donor animals. By comparing ejaculates from boars classified as high- or low-fertility, and evaluating their post-thaw sperm quality, we sought to explore the potential of freezability as a biomarker of reproductive performance.

## 2. Materials and Methods

### 2.1. Ethics Statement

Semen samples were obtained from the commercial artificial insemination (AI) center AIM Ibérica (Topigs Norsvin Spain SLU), located in León, Spain. All procedures were carried out in accordance with relevant European (Directive 2010/63/EU; ES13RS04P, July 2012) and national Spanish regulations (ES300130640127, August 2006) governing the handling and commercialization of AI semen doses, guaranteeing adherence to animal welfare and biosecurity standards.

### 2.2. Animals and Semen Collection

This study used eighteen AI semen doses collected from white pig breeds maintained under standard commercial conditions. Ejaculates were collected using the double-glove method and immediately diluted in a commercial extender (MAGAPOR, Zaragoza, Spain) to a final concentration of 2.4 × 10^9^ spermatozoa per 80 mL dose. Samples were stored at 15–17 °C until further analysis. Each dose corresponded to a single ejaculate from boars with previously confirmed fertility and acceptable semen quality parameters.

### 2.3. Boars Fertility

Fertility data of the boars used in this study were provided by AIM Ibérica (Topigs Norsvin España, León). These data were obtained by using a statistical model developed by Broekhuijse et al. [11], which isolates each boar’s individual contribution to fertility by correcting from confounding factors such as farm conditions, seasonality, and sow-related variables (e.g., parity, repeated farrowings). After this adjustment, fertility outcomes are expressed as standardized deviations from the mean performance of all boars of the same breed within the company. The boars included in this study had fertility data based on more than 250 inseminations per individual (high-fertility boars: ≥ 250; low-fertility boars: ≥ 350). Five parameters were used to characterize fertility performance: total number of piglets born (TNB), stillborn piglets (STB), number of piglets born alive (NBA), farrowing rate (FTR), and gestation length (GLE).

### 2.4. Reagents and Extenders

Unless otherwise specified, all chemicals and reagents were obtained from Sigma-Aldrich (Madrid, Spain). The extenders used throughout the freezing and thawing process included the following:

(i) Cooling extender: Lactose Egg Yolk (LEY), prepared with 80% (*v*/*v*) of 310 mM β-lactose solution and 20% (*v*/*v*) egg yolk (pH 6.0–6.3 and 330–390 mOsm/kg).
(ii) Freezing extender: LEY-Glycerol-Orvus ES Paste (LEYGO), composed of 89.5% LEY, 1.5% Equex-STM, and 9% (*v*/*v*) glycerol (pH of 6.0–6.3 and 2000 mOsm/kg).
(iii) Thawing extender: Beltsville Thawing Solution (BTS) (Minitub Ibérica, Tarragona, Spain) (pH 7.55 and 304 mOsm/kg).

### 2.5. Experimental Design

In this study, 18 commercial doses from 18 different boars were used. The samples were received and analyzed three days after their collection and preparation, during which they were stored at a controlled temperature of 15–17 °C. Each sample was analyzed to assess fresh semen (Fresh-AI Doses) quality parameters, including motility, sperm morphology, and other microscopic variables (described in subsequent sections). An aliquot of each sample was then frozen and thawed. Post-thaw semen quality was assessed immediately after thawing (Post-Thaw) at 37 °C. The parameters analyzed were the same as those for fresh semen, except for sperm morphology.

Objective 1. To assess whether there are differences between fertility groups in fresh (Fresh-AI Doses) and post-thaw (Post-Thaw) semen parameters. To establish experimental groups based on fertility, the five fertility parameters were used: TNB, STB, NBA, FTR, and GLE. A cluster analysis was performed using these data in SPSS (version 25), which allowed for the classification of the animals into two groups: high-fertility (HF) and low-fertility (LF) boars. All semen quality parameters were compared between these groups in fresh and post-thaw conditions, including fertility variables.

Objective 2. Assessment of differences between freezability groups in fresh (Fresh-AI Doses) and post-thaw (Post-Thaw) semen conditions. To establish experimental groups based on freezability, another cluster analysis was performed by using total motility and viability at time 0 as discriminant variables (citation). This analysis also allowed for the classification of the animals into two groups: good freezers (GF) and poor freezers (BF). All semen quality parameters, including fertility variables, were compared between these groups in fresh and post-thaw samples.

Objective 3. Assessment of the effect and the possible interaction between fertility and freezability. To this end, a two-way analysis of variance (two-factor ANOVA) was performed, simultaneously considering fertility (high vs. low) and freezability (good vs. poor) as independent factors. This resulted in four experimental groups: high fertility and good freezability (HF-GF), high fertility and poor freezability (HF-BF), low fertility and good freezability (LF-GF), and low fertility and poor freezability (LF-BF). The effect of each factor was assessed, and all semen quality parameters were compared between the four experimental groups in fresh and post-thaw samples, including fertility variables.

This approach enabled us to evaluate not only the main effects of each factor on semen quality, but also their possible interactions, aiming to identify combinations that could serve as potential predictors of fertility.

### 2.6. Freezing–Thawing Protocol

The freezing protocol followed the methodology described by Martín-San Juan et al., [29]. In brief, 9 mL aliquots of AI doses (approximately 270 × 10^6^ spermatozoa) were centrifuged at 1500× *g* for 5 min. After centrifugation, the supernatant was discarded, and the sperm pellet was resuspended in LEY extender at a 1:2 ratio. The samples were then cooled gradually from 15 °C to 5 °C over a period of 2 h (0.083 °C/min).

Following the cooling phase, spermatozoa were diluted again (1:2) in LEYGO extender and loaded into 0.5 mL PVC-French straws (Minitube International AG, Tiefenbach, Germany), and sealed with polyvinyl alcohol powder. The straws underwent a slow freezing process by being placed 4.5 cm above liquid nitrogen vapors for 20 min in a styrofoam box (~5.25 °C/min), and then plunged into liquid nitrogen (−196 °C) for storage.

Thawing was performed by immersing the straws in a water bath (Lauda Ecoline RE307, Lauda-Königshofen, Germany) at 37 °C for 20 s. Immediately after thawing, sperm samples were diluted 1:1 (*v*/*v*) in BTS extender pre-warmed to 37 °C. Semen quality was then evaluated after thawing.

### 2.7. Sperm Analyses

#### 2.7.1. Sperm Motility and Morphological Abnormalities

Post-thaw samples with high sperm concentration were diluted in BTS extender to achieve a final concentration of 25–30 × 10^6^ spermatozoa/mL and incubated at 37 °C for 10 min prior to analysis. On the other hand, fresh samples were analyzed directly without dilution.

For motility assessment, 5 μL of each sample was placed on a pre-warmed Makler chamber (37 °C) and evaluated using a Computer-Assisted Semen Analysis system (CASA; SPERMTECH^®^ AI Station 1.2.24, Valencia, Spain), connected to a Nikon Eclipse Si RS phase contrast microscope at 100X magnification. At least five non-overlapping video fields were recorded per sample, analyzing a minimum of 200 spermatozoa. Fifty frames per field were captured. Sperm detection thresholds were set at a minimum cell size of 10 μm^2^, a maximum of 69 μm^2^, and a straightness threshold of 50% to classify cells as progressively motile [30]. Sperm kinematic variables, including total sperm motility (MT, %), progressive motility (PM, %), curvilinear velocity (VCL, µm/s), straight-line velocity (VSL, µm/s), average path velocity (VAP, µm/s), percentage of linearity (LIN, %), percentage of straightness (STR, %) and wobble coefficient (WOB, %), were evaluated.

The same CASA system was used to assess sperm morphology in fresh samples. The following parameters were recorded: percentage of morphologically normal spermatozoa (Normal, %), percentage of spermatozoa with proximal cytoplasmic droplets (PD, %), distal droplets (DDs, %), and tail abnormalities (TAs, %). Additionally, the system provided the percentage of total motile spermatozoa with normal morphology, referred to as total normal sperm motility (MNT, %).

#### 2.7.2. Fluorescence Microscopy

Fluorescence microscopic analysis was performed using a Zeiss Axio Observer microscope (ZEISS, Oberkochen, Germany) equipped with an LED illumination system (Colibri 5, ZEISS, Oberkochen, Germany) that includes UV (385 nm), blue (475 nm), green (555 nm), and far-red/near-infrared (647 nm) excitation lines for bright-field and epifluorescence imaging. Image acquisition and analysis were managed using ZEN software (version 3.9.4) from ZEISS, facilitating the simultaneous visualization of multiple fluorescence channels.

Staining solutions were freshly prepared from stock solutions of each fluorochrome at the appropriate working concentrations. For each staining procedure, 50 µL of the samples was stained with 5 µL of each staining solution and incubated 10 min at 37 °C in darkness. A minimum of 200 spermatozoa per treatment were analyzed in all staining.

##### Viability and Acrosome Integrity (PI/PNA)

Membrane integrity and acrosome damage were evaluated by fluorescence microscopic employing propidium iodide (PI) and fluorescein isothiocyanate-conjugated peanut (*Arachis hypogaea*) agglutinin (PNA-FITC) [29]. From stock solutions of PI (10 mg/mL, Life Technologies Corporation) and PNA (1 mg/mL, Life Technologies Corporation), staining solutions with concentrations of 0.4 mM PNA and 0.4 mg/mL PI were prepared. Four populations were recorded: spermatozoa with undamaged membrane and intact acrosome (Live, non-reacted Acr, %), undamaged membrane and reacted acrosome (Live, reacted Acr, %), damaged membrane and intact acrosome (Dead, non-reacted Acr, %), and damaged membrane and reacted acrosome (Dead, reacted Acr, %).

##### Mitochondrial Membrane Potential (JC-1)

Mitochondrial membrane potential (Δψm) was assessed using the fluorescent probe JC-1 and visualized under fluorescence microscopy [30]. A working solution at 0.04 mM was prepared from a 1 mM JC-1 stock solution (Life Technologies Corporation, Zaragoza, Spain). JC-1 exists mainly as a green fluorescent monomer (530 nm), but under high mitochondrial membrane potential it forms aggregates that emit orange-red fluorescence (590 nm). The percentage of sperm displaying high membrane potential (JC-1 orange) was determined.

##### Mitochondrial Activation

Mitochondrial activity was evaluated using Mitotracker Deep Red (MTDR) staining, following the method described by Sargiacomo et al. [31]. This far-red fluorescent probe selectively accumulates in the mitochondria of viable cells, with active mitochondria exhibiting stronger fluorescence emission (665 nm) compared to inactive or damaged ones. From stock solutions of YO-PRO-1 (1 mM, Thermo Fisher Scientific, Madrid, Spain), PI (10 mg/mL), and MTDR (2.5 µg/µL, Thermo Fisher Scientific), staining solutions with concentrations of 0.02 mM YO-PRO-1, 0.4 mg/mL PI, and 0.1 µg/µL MTDR were prepared. Four populations were recorded: live spermatozoa with active mitochondria (Live, MITOact, %), live spermatozoa with inactive mitochondria (Live, MITOdeact, %), dead spermatozoa with active mitochondria (Dead, MITOact, %) and dead spermatozoa with inactive mitochondria (Dead, MITOdeact, %).

##### Apoptotic-like Changes (Early Membrane Destabilization)

YO-PRO-1 staining was employed to detect spermatozoa exhibiting early membrane destabilization [32]. This dye selectively permeates apoptotic cells and binds to DNA, resulting in green fluorescence emission at 509 nm. From stock solutions of YO-PRO-1 (1 mM) and PI (10 mg/mL), staining solutions with concentrations of 0.02 mM YO-PRO-1 and 0.4 mg/mL PI were prepared. Four populations were recorded: live non-apoptotic spermatozoa (Live, non-Apoptotic, %), live apoptotic spermatozoa (Live, Apoptotic, %), dead non-apoptotic spermatozoa (Dead, non-Apoptotic, %) and dead apoptotic spermatozoa (Dead, Apoptotic, %).

##### Oxidative Stress

Dihydroethidium (DHE) staining was used to evaluate cellular oxidative stress. This probe reacts with cytosolic superoxide radicals, becoming oxidized and subsequently intercalating with DNA, which leads to red fluorescence emission at 606 nm (Zielonka and Kalyanaraman, 2010) [33]. From stock solutions of YO-PRO-1 (1 mM) and dihydroethidium (1 mM, Thermo Fisher Scientific), staining solutions with concentrations of 0.02 mM YO-PRO-1 and 0.5 mg/mL DHE were prepared. Four populations were recorded: non-apoptotic and non-oxidized spermatozoa (Non-Apoptotic, Non-Oxi, %), non-apoptotic and oxidized spermatozoa (Non-Apoptotic, Oxi, %), apoptotic and non-oxidized spermatozoa (Apoptotic, Non-Oxi, %), and apoptotic and oxidized spermatozoa (Apoptotic, Oxi, %).

### 2.8. Statistical Analysis

All datasets were first tested for normality and homogeneity of variances. To classify ejaculates according to fertility or freezability, cluster analysis was performed using SPSS (version 25, IBM, Armonk, NY, USA), applying group means as the classification criterion. The distance between clusters was computed using the squared Euclidean distance, defined as the sum of the squared differences in values across observations within each group. For the fertility-based classification, cluster analysis included the following variables: TNB, STB, NBA, FTR, and GLE. For the classification based on freezability, the percentage of total motile sperm and the percentage of spermatozoa with undamaged membrane and intact acrosome post-thawing were used, following criteria previously validated in the literature for the classification of ejaculates in terms of freezability [34,35]. Differences between the groups (fertility or freezability) were evaluated for all measured variables by using the Student’s *t*-test, considering *p* < 0.05 as the threshold for statistical significance.

To evaluate the main effects and interactions between fertility and freezability, a two-way ANOVA was conducted using GraphPad Prism version 10.2.3 (GraphPad Software, Boston, MA, USA), including both variables as fixed factors. Post hoc comparisons were analyzed using Tukey’s test (*p* < 0.05). In addition, for each comparison (fertility groups, freezability groups, and the four combined groups in the two-way ANOVA), potential differences between the two time points (Fresh-Doses AI (Fresh) and post-thawing (Thaw)) were evaluated within each group. Data are expressed as the mean ± standard error of the mean (SEM), unless otherwise indicated.

## 3. Results

### 3.1. Differences in Semen Parameters Between Fertility Groups (Objective 1)

Cluster analysis based on fertility-related variables classified the ejaculates into two groups (high-fertility, HF (*n*: 7); low-fertility, LF (*n*: 11)). Only the number of piglets born alive (NBA) and farrowing rate (FTR) significantly differed between groups (*p* = 0.036 and *p* < 0.0001, respectively; Table 1).

**Figure 1:**
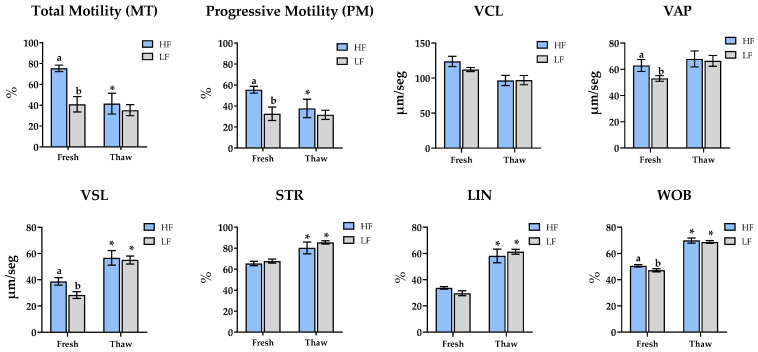
Sperm motility and kinematic analysis in Fresh-Doses AI and thawing samples. HF: high-fertility (blue bar), LF: low-fertility (gray bar). Total sperm motility (MT, %), progressive motility (PM, %), curvilinear velocity (VCL, μm/s), straight-line velocity (VSL, μm/s), average path velocity (VAP, μm/s), percentage of linearity (LIN, %), percentage of straightness (STR, %), and wobble coefficient (WOB, %). Significant differences (*p* < 0.05) between HF and LF at fresh or thaw samples are in-dicated with a superscript lowercase letter (a, b). * in thaw samples indicates significant differences between fresh and thaw samples (*p* < 0.05). Data are depicted as the means ± SEM.

No significant differences in sperm morphological abnormalities were found between fertility groups, except for the percentage of motile spermatozoa without morphological abnormalities (total normal motility, MNT), which was significantly lower in the LF group (*p* < 0.05) (Table 1). This trend was consistent with the motility data obtained from fresh semen, where LF ejaculates showed reduced values for MT (75.4 ± 3.1 vs. 40.9 ± 7.3), PM (55.5 ± 3.3 vs. 32.6 ± 6.4), VAP (62.9 ± 4.6 vs. 53 ± 2.1), VSL (38.7 ± 2.8 vs. 28.4 ± 2.6), and WOB (50.6 ± 0.9 vs. 47.2 ± 1.1) compared to the HF group. No differences in motility were observed between fertility groups in the post-thaw samples (Figure 1).

When evaluating the effect of cryopreservation within each fertility group, MT and PM were significantly reduced in the HF group (75.4 ± 3.1 vs. 41.5 ± 10 and 55.5 ± 3.3 vs. 37.7 ± 8.8, respectively) but remained stable in LF (40.9 ± 7.3 vs. 35.2 ± 5.3 and 32.6 ± 6.4 vs. 37.7 ± 4.4, respectively). Conversely, both groups exhibited an increase in VSL, STR, LIN, and WOB post-thawing.

**Figure 2:**
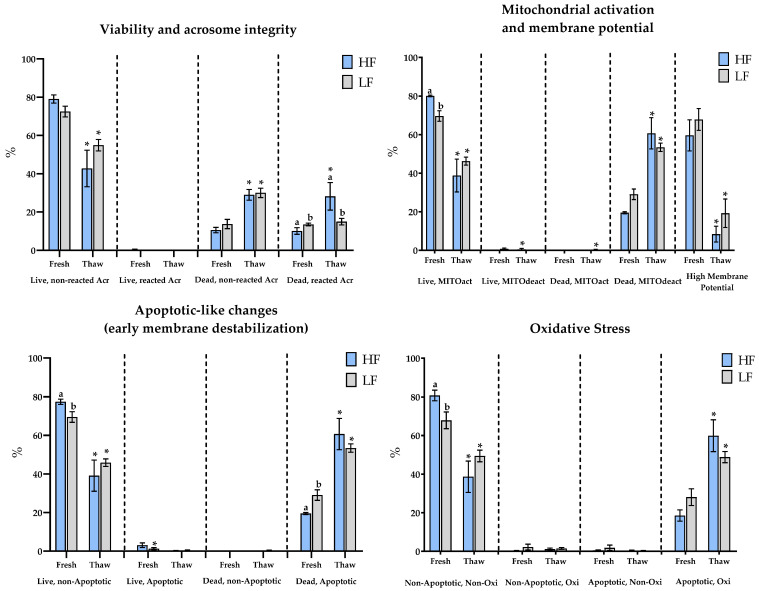
Fluorescence microscopic analysis in Fresh-Doses AI and thawing samples. HF: high-fertility (blue bar), LF: low-fertility (gray bar). Live, non-reacted Acr (%): spermatozoa with undamaged membrane and intact acrosome; Live, reacted Acr (%): undamaged membrane and reacted acrosome; Dead, non-reacted Acr (%): damaged membrane and intact acrosome; Dead, reacted Acr (%): damaged membrane and reacted acrosome; Live, MITOact (%): live spermatozoa with active mitochondria; Live, MITOdeact, (%): live spermatozoa with inactive mitochondria; Dead, MITOact (%): dead spermatozoa with active mitochondria; Dead, MITOdeact (%): dead spermatozoa with inactive mitochondria; high membrane potential: percentage of sperm displaying high membrane potential (JC-1 orange); Live, non-Apoptotic (%): live non-apoptotic spermatozoa; Live, Apoptotic (%): live apoptotic spermatozoa; Dead, non-Apoptotic (%): dead non-apoptotic spermatozoa; Dead, Apoptotic (%): dead apoptotic spermatozoa; Non-Apoptotic, Non-Oxi (%): non-apoptotic and non-oxidized spermatozoa; Non-Apoptotic, Oxi (%): non-apoptotic and oxidized spermatozoa; Apoptotic, Non-Oxi (%): apoptotic and non-oxidized spermatozoa; and Apoptotic, Oxi (%): apoptotic and oxidized spermatozoa. Significant differences (*p* < 0.05) between HF and LF at fresh or thaw samples are indicated with a superscript lowercase letter (a, b). * in thaw samples indicates significant differences between fresh and thaw samples (*p* < 0.05). Data are depicted as the means ± SEM.

Sperm viability and sublethal damage revealed apparent fertility-group-related differences in fresh semen. The LF group displayed significantly higher percentages of dead spermatozoa with reacted acrosome (10.6 ± 1.4 vs. 13.7 ± 2.4) and apoptotic death (19.5 ± 0.5 vs. 29.1 ± 2.7), while exhibiting lower percentages of viable non-apoptotic cells (77.4 ± 1.3 vs. 69.5 ± 2.7), sperm with active mitochondria (80.1 ± 0.4 vs. 69.6 ± 2.7), and non-apoptotic non-oxidized sperm (80.8 ± 2.7 vs. 67.9 ± 4.3) compared to the HF group (*p* < 0.05 for all comparisons; Figure 2). However, post-thaw comparisons between groups revealed only one significant difference: the HF group exhibited a higher percentage of dead sperm with reacted acrosome (28.2 ± 7.16 vs. 15 ± 1.7).

Cryopreservation led to marked changes in all fluorescence-based parameters across both fertility groups. In both HF and LF, a significant decline was observed in viable spermatozoa, including viable non-apoptotic (77.4 ± 1.36 vs. 39.1 ± 8.1 and 69.5 ± 2.7 vs. 45.9 ± 1.9, respectively), non-oxidized (80.8 ± 2.7 vs. 38.7 ± 8.1 and 67.9 ± 4.3 vs. 49.4 ± 3, respectively), and mitochondrial-active cells (80.1 ± 0.4 vs. 38.81 ± 8.5 and 69.6 ± 2.7 vs. 46.2 ± 2.1, respectively), as well as those with high mitochondrial membrane potential (59.6 ± 8 vs. 8.4 ± 4.1 and 67.8 ± 5.6 vs. 19.2 ± 7.4, respectively). Simultaneously, there was a significant increase in dead sperm with reacted acrosome (10.1 ± 1.7 vs. 28.2 ± 7.2 and 13.5 ± 0.7 vs. 15 ± 1.7, respectively), apoptotic cells (19.5 ± 0.5 vs. 60.7 ± 8.1 and 29.1 ± 2.7 vs. 53.4 ± 2.2, respectively), mitochondrial-inactive sperm (18.8 ± 0.7 vs. 60.5 ± 8.1 and 25.6 ± 2 vs. 53.7 ± 2, respectively), and oxidized apoptotic cells (18.5 ± 2.9 vs. 59.9 ± 8.2 and 28.1 ± 4.3 vs. 48.8 ± 2.9, respectively). Notably, only in the HF group, the proportion of dead sperm with reacted acrosome increased significantly, alongside a significant reduction in viable apoptotic sperm post-thaw.

### 3.2. Differences in Semen Parameters Between Freezability Groups (Objective 2)

Cluster analysis based on post-thaw sperm quality parameters classified the ejaculates into two distinct freezability groups: good freezers (GF, *n* = 10) and bad freezers (BF, *n* = 8). As expected, the classification variables—percentage of live spermatozoa and percentage of total motile sperm post-thaw—differed significantly between groups (*p* < 0.05), with GF exhibiting substantially higher values (59.0% vs. 39.2% live sperm and 54.0% vs. 17.3% total motility, respectively).

No significant differences were observed between GF and BF groups in fertility traits (TNB, STB, NBA, FTR, and GLE; Table 2) or in the incidence of sperm morphological abnormalities. However, the proportion of motile sperm without morphological defects was significantly higher in the GF group (*p* < 0.05), consistent with differences observed in sperm motility.

**Figure 3:**
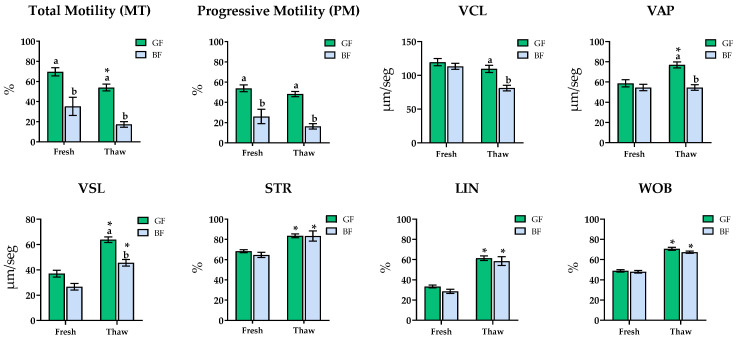
Sperm motility and kinematic analysis in Fresh-Doses AI and thawing samples. GF: good freezers (green bar), BF: bad freezers (blue bar). Total sperm motility (MT, %), progressive motility (PM, %), curvilinear velocity (VCL, μm/s), straight-line velocity (VSL, μm/s), average path velocity (VAP, μm/s), percentage of linearity (LIN, %), percentage of straightness (STR, %), and wobble coefficient (WOB, %). Significant differences (*p* < 0.05) between HF and LF for fresh or thaw samples are indicated with a superscript lowercase letter (a, b). * in thaw samples indicates significant differences between fresh and thaw samples (*p* < 0.05). Data are depicted as means ± SEM.

Fresh semen from GF animals exhibited higher motility parameters (MT (69.6 ± 4.2 vs. 35.1 ± 9.1), PM (53.9 ± 3.3 vs. 26.1 ± 7.1), and VSL (37 ± 2.7 vs. 26.6 ± 2.6)) compared to BF. These differences were also presented post-thaw, with GF samples showing significantly greater values for MT, PM, VCL, VAP, and VSL (*p* < 0.05) (Figure 3).

Interestingly, cryopreservation affected the groups differently: only GF ejaculates showed a significant decline in MT (69.6 ± 4.2 vs. 54 ± 3.3) and an increase in VAP (58.7 ± 3.6 vs. 77 ± 3) between fresh and post-thaw samples. Regardless of group, both exhibited post-thaw increases in VSL (GF: 37 ± 2.7 vs. 63.8 ± 2.2; BF: 26.6 ± 2.6 vs. 45.3 ± 2.3), STR (GF: 68.4 ± 1.5 vs. 83.6 ± 1.9; BF: 64.7 ± 2.6 vs. 83.4 ± 5), LIN (GF: 33.4 ± 1.3 vs. 61.4 ± 2.3; BF: 28.5 ± 2.1 vs. 58.5 ± 4.4), and WOB (GF: 48.9 ± 1.2 vs. 70.7 ± 1.4; BF: 48 ± 1.3 vs. 67.3 ± 1.1).

**Figure 4:**
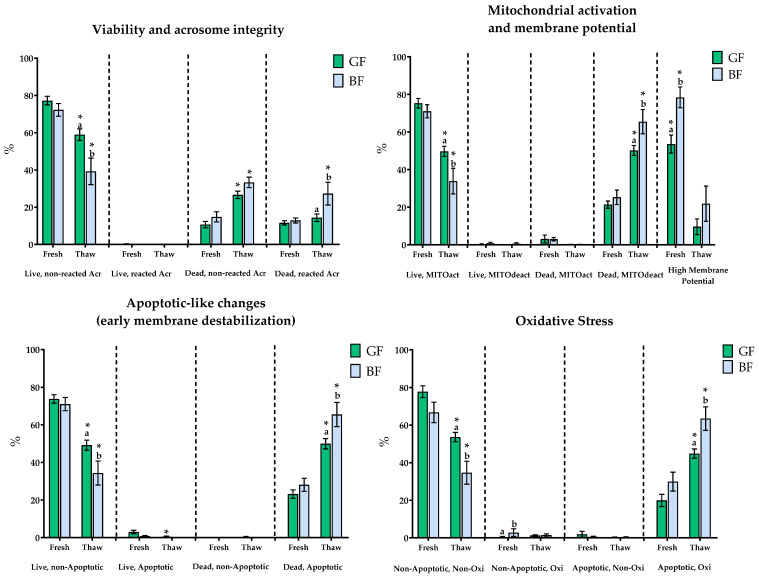
Fluorescence microscopic analysis in Fresh-Doses AI and thawing samples. GF: good freezers (green bar), BF: bad freezers (blue bar). Live, non-reacted Acr (%): spermatozoa with undamaged membrane and intact acrosome; Live, reacted Acr (%): undamaged membrane and reacted acrosome; Dead, non-reacted Acr (%): damaged membrane and intact acrosome; Dead, reacted Acr (%): damaged membrane and reacted acrosome; Live, MITOact (%): live spermatozoa with active mitochondria; Live, MITOdeact, (%): live spermatozoa with inactive mitochondria; Dead, MITOact (%): dead spermatozoa with active mitochondria; Dead, MITOdeact (%): dead spermatozoa with inactive mitochondria; high membrane potential: percentage of sperm displaying high membrane potential (JC-1 orange); Live, non-Apoptotic (%): live non-apoptotic spermatozoa; Live, Apoptotic (%): live apoptotic spermatozoa; Dead, non-Apoptotic (%): dead non-apoptotic spermatozoa; Dead, Apoptotic (%): dead apoptotic spermatozoa; Non-Apoptotic, Non-Oxi (%): non-apoptotic and non-oxidized spermatozoa; Non-Apoptotic, Oxi (%): non-apoptotic and oxidized spermatozoa; Apoptotic, Non-Oxi (%): apoptotic and non-oxidized spermatozoa; and Apoptotic, Oxi (%): apoptotic and oxidized spermatozoa. Significant differences (*p* < 0.05) between HF and LF for fresh or thaw samples are indicated with a superscript lowercase letter (a, b). * In thaw samples indicate significant differences between fresh and thaw (*p* < 0.05). Data are depicted as means ± SEM.

Fluorescence-based sperm quality assessment revealed that fresh semen showed differences only in mitochondrial membrane potential (53.6 ± 4.8 vs. 78 ± 5.5) and the proportion of non-apoptotic oxidized sperm (0.4 ± 0.3 vs. 2.8 ± 2), with higher values for both parameters in the BF group (*p* < 0.05). In contrast, post-thaw samples revealed marked differences: GF samples had significantly higher proportions of viable sperm with intact acrosomes (GF: 59 ± 3.1 vs. BF: 39.2 ± 7.1), non-apoptotic viable sperm (GF: 49.2 ± 2.7 vs. BF: 34.4 ± 6.4), sperm with active mitochondria (GF: 49.7 ± 2.7 vs. BF: 33.8 ± 6.8), and non-oxidized non-apoptotic sperm (GF: 53.7 ± 2.5 vs. BF: 34.7 ± 6.1) (*p* < 0.05). Meanwhile, BF samples exhibited significantly higher percentages of dead sperm with reacted acrosome (GF: 14.4 ± 2 vs. BF: 27.3 ± 6.1), apoptotic dead sperm (GF: 49.7 ± 2.7 vs. BF: 33.8 ± 6.8), sperm without mitochondrial activity (GF: 50.2 ± 2.6 vs. BF: 65.5 ± 6.4), and oxidized apoptotic sperm (GF: 44.9 ± 2.5 vs. BF: 63.5 ± 6.3) (Figure 4).

Intra-group comparisons between fresh and post-thaw samples revealed significant alterations across nearly all parameters. Both groups showed a decline in viable sperm populations and an increase in damaged and apoptotic subpopulations. However, the proportion of dead sperm with reacted acrosome remained unchanged in the GF group post-thaw (11.6 ± 1.1 vs. 14.4 ± 2).

### 3.3. Combined Effects of Fertility and Freezability on Semen Parameters (Objective 3)

To investigate potential combined effects of fertility and freezability, animals were categorized into four groups based on their classification from previous cluster analyses: high-fertility and good freezability (HF-GF, *n* = 5), high-fertility and poor freezability (HF-BF, *n* = 2), low-fertility and good freezability (LF-GF, *n* = 5), and low-fertility and poor freezability (LF-BF, *n* = 6).

Analysis of fertility parameters revealed a significant effect of fertility on the farrowing rate (FTR) (*p* < 0.05). The HF-GF group exhibited significantly higher FTR compared to LF-GF and LF-BF, while no differences were found between HF-GF and HF-BF (Table 3).

As previously observed, no significant differences were found among the four groups in terms of total morphological abnormalities. However, the percentage of MNT was significantly influenced by both fertility and freezability (*p* < 0.05), with no interaction. The LF-BF group showed the lowest figure compared to all other groups (Table 4).

Analysis of sperm motility in fresh semen revealed significant differences in several parameters. The LF-BF group showed lower MT values compared to the other groups (*p* < 0.05), with significant main effects of fertility, freezability, and their interaction. For PM, significant effects of fertility and freezability were found, though no interaction was observed. VSL was affected solely by fertility, with HF-GF outperforming LF-BF. In post-thaw samples, freezability emerged as the dominant factor: both BF groups, regardless of fertility, exhibited significantly lower values for MT, PM, VCL, VAP, and VSL compared to GF groups. A significant interaction between fertility and freezability was identified for PM (*p* < 0.05). No differences were found between groups for LIN, STR, or WOB (Table 5).

Fluorescence-based semen quality analysis in fresh samples revealed no significant group differences across any variable. However, apoptotic dead sperm and viable sperm with active mitochondria were affected by fertility, while mitochondrial membrane potential was influenced by freezability. Nonetheless, these effects did not translate into statistically significant differences between groups. Post-thaw samples, however, showed consistent and marked differences. Almost all evaluated parameters exhibited significant effects of fertility, freezability, and their interaction. The HF-BF group had the lowest percentages of viable sperm with intact acrosomes, viable apoptotic sperm, and viable sperm with or without mitochondrial activity (*p* < 0.05). This group also had the highest levels of dead sperm with reacted acrosome, apoptotic dead sperm, sperm without mitochondrial activity, and oxidized apoptotic sperm. Additionally, non-oxidized viable sperm were significantly lower in HF-BF and LF-BF compared to LF-GF, with HF-GF also differing from HF-BF (Table 6).

## 4. Discussion

Currently, pig production faces a major challenge: the early identification of subfertile animals. These animals are often not detected until they have been in the production system for a long time, leading to significant economic losses for producers [1,2,3]. Reliable biomarkers for detecting subfertility in boars are still under investigation, and although some studies have shown promising results [1], it is necessary to develop practical and reliable tools that enable efficient selection and rapid identification.

In this context, semen freezability is proposed as a possible complementary test to detect subfertile boars. The underlying hypothesis is that spermatozoa with greater resistance to cryopreservation-induced damage may also reflect better functional competence during fertilization, both natural and assisted. This makes sense considering the performance of frozen semen in artificial insemination in pigs. Artificial insemination with frozen semen generally yields poorer fertility results and lower litter sizes than when using fresh or refrigerated semen [36]. This is often attributed to cryodamage during freezing, which leads to reduced sperm quality and an average loss in cell viability of 50% [35]. However, this does not fully explain the poorer reproductive outcomes, as it could theoretically be overcome by increasing the number of frozen sperm used. Yet, increasing sperm concentration in frozen doses has shown contradictory results, and does not always improve fertility outcomes [37,38,39].

This suggests that cryopreservation may reveal hidden structural or functional defects in sperm beyond basic viability, possibly related to sperm structure or composition. Recently, it has been established that there is a difference in membrane composition between good and poor freezers [25]. This different composition could not only explain the variation in freezability, but also differences in fertility, as membrane resistance and interaction capacity with the external environment may influence fertility. Therefore, cryopreservation acts as an extreme physiological challenge, subjecting cells to mechanical, osmotic, and oxidative changes that may selectively damage sperm with structural or functional undetectable weaknesses by routine techniques, potentially related to fertility.

Our results reveal a complex relationship between fresh semen quality, resistance to cryopreservation, and recorded fertility. While differences in fresh semen quality parameters were observed between high-fertility (HF) and low-fertility (LF) boars, these differences did not directly translate into a clear relationship between freezability (evaluated post-thaw) and the predefined fertility groups.

Despite previous findings indicating that fertility cannot be predicted by routine semen quality analysis (reviewed by Jung et al. [40]), our study shows that after classifying boars by fertility, significant differences were found between groups in terms of fresh semen (insemination AI dose) parameters, but only minimal differences were observed in post-thaw parameters. Our results show clear differences in terms of motility between fertility groups, with lower total and progressive motility, and lower proportions of morphologically normal motile sperm, along with reduced VAP and VSL in the LF group. This aligns with earlier findings where motility below 60% was associated with reduced fertility [13], or studies such as Tardif et al. [41], who identified sperm motility as one of the most important indicators of in vivo fertility. Collins et al. [42] also found that motility could serve as a fertility predictor when combined with other parameters such as zona-binding ability, normal morphology, acrosome integrity, and the presence of distal droplets. It is true that our results showed lower values than typically expected for commercial semen doses, particularly in the low-fertility group. However, the samples were not analyzed until three days after collection and dilution due to logistical and transport limitations between the farm and the laboratory. It is also possible that the prolonged storage period contributed to the observed differences in motility. Similar observations have been reported by Ruiz-Sanchez et al. [43], who found significant correlations between the motility of extended boar semen after several days of storage and fertility outcomes, and by Foxcroft et al. [44], who proposed that evaluating sperm motility after storage is a suitable method with which to predict boar fertility. Therefore, as we observed in our study, LF animals would show a continually shorter shelf life than GF males the longer the storage period.

Fluorescence microscopy analysis revealed greater sublethal damage in fresh semen from LF boars, including higher percentages of dead sperm with reacted acrosomes, increased apoptotic death, and lower percentages of viable, non-apoptotic cells with active and non-oxidized mitochondria. This supports the idea that while conventional parameters may fail to detect fertility-related differences [11,13], more detailed analysis of fresh sperm functionality using techniques like flow cytometry or fluorescence microscopy [9,12] can reveal underlying fertility-associated differences. Additionally, our study is based on boar fertility classification using reproductive records of over 250 inseminations, compared to the whole company’s population, while other studies have used fewer inseminations or animals, and relied on punctual in vivo or in vitro fertility values [45,46,47], which is also a source of variation.

However, many of the fertility-related differences observed in fresh semen between HF and LF groups disappeared after cryopreservation. Although the freeze–thaw process induced significant damage in both groups (reduced viability, increased acrosomal damage, apoptosis, etc.), no clear differences were found in post-thaw quality between fertility groups, except for a higher percentage of dead sperm with reacted acrosomes in the HF group. This may still relate to fertility, since Daigneault et al. [28] indicated that some post-thaw parameters, like acrosome-damaged sperm or total motility, could predict ejaculate fertility. Furthermore, total and progressive motility decreased significantly post-thaw in the HF group only, which may suggest that LF sperm were already compromised or respond differently to freezing.

As expected, the analysis based on freezability (Objective 2) showed that good freezers (GF) had significantly better post-thaw quality across almost all parameters evaluated (motility, viability, acrosome integrity, apoptotic state, mitochondrial activity, and oxidative stress).

However, we could not identify any single fresh semen parameter that reliably predicted boars’ final freezability. As with subfertile animals, there is currently no feasible way to identify boars as good or poor freezers beforehand. Our results agree with other studies showing that conventional sperm parameters in fresh/refrigerated semen cannot predict the ability of an ejaculate to withstand cryopreservation [45,48,49]. However, in fresh semen, GF boars showed higher motility (MNT, MT, PM, and VSL) and lower membrane potential (*p* < 0.05). Researchers studying different species have found that pre-freezing motility may predict or strongly relate to future freezability [50,51,52], and some have even suggested that high VSL values could be a good predictor of freezability [53], which aligns with our results.

On the other hand, no significant differences were found in fertility parameters (TNB, STB, NBA, FTR, and GLE) between the two freezability groups. This may suggest that the ability of an ejaculate to resist cryopreservation alone is not a direct predictor of fertility, supporting the idea that fertility is a complex multifactorial trait that cannot be reduced to cold resistance [1,2]. However, for Objective 3, significant effects were found for fertility, freezability, and their interaction on post-thaw parameters such as Live non-reacted Acr, Dead reacted Acr, Live non-Apoptotic, Dead Apoptotic, Live MITOdeact, Live MITOact, Dead MITOdeact, Non-apoptotic non-oxidized, and Apoptotic oxidized. In most parameters, the HF-BF group differed from the others, with worse post-thaw quality values than the LF-BF group. These results suggest that although not all high-fertility animals can be identified by using the freezability test, a few animals with very poor freezability may still have high fertility. Very few studies have explored a potential relationship between fertility and freezability. Selles et al. [47] concluded that freezability can predict in vitro fertilization but not normal insemination fertility, although their study only included five boars. Gil et al. [46] found a relationship between good freezers and better in vitro fertility parameters, but not all boars classified as “good” freezers achieved high in vitro fertility, while some “poor” freezers reached acceptable fertility rates. These findings are consistent with ours, suggesting the presence of good freezers with low fertility and poor freezers with good fertility. Casas et al. [45] conducted in vivo inseminations with semen from good and poor freezers and found that good freezers had higher fertility, with no differences compared to refrigerated semen, but pregnancy and farrowing rates were below 60%, suggesting that not all inseminations with good freezer animals were equally successful—possibly due to the presence of good freezers with low fertility.

Finally, we observed that total motility in fresh semen was the only parameter consistently affected by both fertility and freezability. Boars in the LF–PF group had the lowest values, which suggests that this variable could be particularly informative.

## 5. Conclusions

In conclusion, our results indicated that a freezability test may serve as an initial indicator of future fertility, where low motility in fresh semen is associated with low fertility, and poor resistance to freezing, with particularly low post-thaw semen quality parameters (except motility), could indicate a population that may actually have good fertility. Although further studies and larger sample sizes are needed, our results can be considered robust as the boars were evaluated based on fertility after more than 250 inseminations. Additionally, it is not necessary to assess the freezability of each ejaculate used to determine fertility, as freezability has been shown to be an intrinsic characteristic of the animal [35,54,55].

## Figures and Tables

**Table 1:** Fertility and sperm morphology parameters in Fresh-Doses AI in boar classified as high- and low-fertility.

	Boar Fertility				Sperm Morphology		
	HF	LF	*p*-Value		HF	LF	*p*-Value
TNB	0.5 ± 0.05	0.25 ± 0.09	0.06	MNT, %	69 ± 3.9	36.7 ± 7.1	0.003
STB	0.003 ± 0.03	0.08 ± 0.05	0.28	Normal, %	89.7 ± 2	89.5 ± 2	0.93
NBA	0.49 ± 0.04	0.17 ± 0.09	0.036	PD, %	3.9 ± 0.9	5.1 ± 2.3	0.21
FTR	2.98 ± 0.5	−0.19 ± 0.27	<0.0001	DDs, %	4.7 ± 1.1	3.5 ± 0.5	0.28
GLE	0.11 ± 0.1	0.17 ± 0.15	0.78	TAs, %	0.03 ± 0.01	0.08 ± 0.01	0.69

HF: high-fertility, LF: low-fertility. Total number of piglets born (TNB), stillborn piglets (STB), number of piglets born alive (NBA), farrowing rate (FTR), and gestation length (GLE). Total normal sperm motility (MNT, %), normal spermatozoa (Normal, %), spermatozoa with proximal cytoplasmic droplets (PD, %), distal droplets (DDs, %), and tail abnormalities (TAs, %). Data are depicted as means ± SEM.

**Table 2:** Fertility and sperm morphology parameters in Fresh-Doses AI in boar classified as good or bad freezers.

	Boar Fertility				Sperm Morphology		
	GF	BF	*p*-Value		GF	BF	*p*-Value
TNB	0.39 ± 0.09	0.3 ± 0.1	0.55	MNT, %	63.2 ± 4.9	31.9 ± 8.5	0.0041
STB	0.002 ± 0.02	0.1 ± 0.06	0.21	Normal, %	89.3 ± 2.2	89.9 ± 1.7	0.84
NBA	0.38 ± 0.08	0.2 ± 0.11	0.12	PD, %	5.5 ± 2.4	3.6 ± 1.3	0.63
FTR	1.46 ± 0.7	0.51 ± 0.43	0.31	DDs, %	3.5 ± 0.8	4.7 ± 0.7	0.12
GLE	0.24 ± 0.16	0.03 ± 0.11	0.32	TAs, %	0.05 ± 0.03	0.07 ± 0.04	0.94

GF: good freezers, BF: bad freezers. Total number of piglets born (TNB), stillborn piglets (STB), number of piglets born alive (NBA), farrowing rate (FTR), and gestation length (GLE). Total normal sperm motility (MNT, %), normal spermatozoa (Normal, %), spermatozoa with proximal cytoplasmic droplets (PD, %), distal droplets (DDs, %), and tail abnormalities (TAs, %). Data are depicted as means ± SEM.

**Table 3:** Fertility parameters in Fresh-Doses AI in boar classified by fertility and freezability.

	HF-GF	HF-BF	LF-GF	LF-BF	*p*-Value Interaction	*p*-Value Freezability	*p*-Value Fertility
TNB	0.52 ± 0.07	0.45 ± 0.09	0.25 ± 0.16	0.25 ± 0.12	0.8025	0.8194	0.1217
STB	0.02 ± 0.03	−0.04 ± 0.02	−0.02 ± 0.03	0.16 ± 0.07	0.0787	0.4091	0.2231
NBA	0.49 ± 0.04	0.49 ± 0.11	0.27 ± 0.13	0.09 ± 0.12	0.5189	0.5082	0.0281
FTR	3.35 ± 0.63 ^a^	2.03 ± 0.02 ^ab^	−0.44 ± 0.39 ^b^	0.009 ± 0.38 ^b^	0.1269	0.4378	0.0001
GLE	0.22 ± 0.15	−0.16 ± 0.11	0.26 ± 0.3	0.09 ± 0.14	0.6648	0.264	0.5364

HF-GF: high-fertility and good freezability, HF-BF: high-fertility and poor freezability, LF-GF: low-fertility and good freezability, and LF-BF: low-fertility and poor freezability. Total number of piglets born (TNB), stillborn piglets (STB), number of piglets born alive (NBA), farrowing rate (FTR), and gestation length (GLE). Significant differences (*p* < 0.05) between groups are indicated with a superscript lowercase letter (a, b). Data are depicted as means ± SEM.

**Table 4:** Sperm morphology parameters in Fresh-Doses AI in boar classified by fertility and freezability.

	HF-GF	HF-BF	LF-GF	LF-BF	*p*-Value Interaction	*p*-Value Freezability	*p*-Value Fertility
MNT, %	71.3 ± 4.4 ^a^	63.4 ± 8.8 ^a^	55 ± 7.6 ^a^	21.4 ± 6.4 ^b^	0.1066	0.0147	0.0016
Normal, %	90 ± 2.2	89 ± 6	88.6 ± 4.2	90.2 ± 1.6	0.7012	0.9399	0.9786
PD, %	4.1 ± 1.2	3.5 ± 1.1	6.9 ± 4.8	3.6 ± 1.7	0.7054	0.5631	0.6659
DDs, %	4.6 ± 1.3	5.1 ± 2.8	2.4 ± 0.5	4.5 ± 0.6	0.4872	0.2515	0.2338
TAs, %	0.02 ± 0.008	0.05 ± 0.05	0.09 ± 0.05	0.08 ± 0.05	0.7013	0.8166	0.402

HF-GF: high-fertility and good freezability, HF-BF: high-fertility and poor freezability, LF-GF: low-fertility and good freezability, LF-BF: low-fertility and poor freezability. Total normal sperm motility (MNT, %), normal spermatozoa (Normal, %), spermatozoa with proximal cytoplasmic droplets (PD, %), distal droplets (DDs, %), and tail abnormalities (TAs, %). Significant differences (*p* < 0.05) between groups are indicated with a superscript lowercase letter (a, b). Data are depicted as means ± SEM.

**Table 5:** Sperm motility parameters in fresh-Doses AI and thawing samples in boar classified by fertility and freezability.

		HF-GF	HF-BF	LF-GF	LF-BF	*p*-Value Interaction	*p*-Value Freezability	*p*-Value Fertility
MT, %	Fresh	77.6 ± 3.7 ^a^	69.9 ± 5 ^a^	61.6 ± 5.7 ^a^	23.6 ± 6.7 ^b^	0.0397	0.0041	0.0004
	Thaw	55.2 ± 6.4 ^a^	7.4 ± 6.7 ^b^	52.7 ± 3.1 ^a^	20.6 ± 1.6 ^b^	0.1183	<0.0001	0.2736
PM, %	Fresh	57.9 ± 2 ^a^	49.6 ± 11.7 ^a^	49.91 ± 6.1 ^a^	18.2 ± 6 ^b^	0.0907	0.0077	0.0085
	Thaw	50.1 ± 4.8 ^a^	6.7 ± 6.3 ^b^	46.3 ± 2.3 ^a^	19.5 ± 1.6 ^a^	0.0415	<0.0001	0.2495
VCL, µm/s	Fresh	125 ± 9.6	121 ± 14.4	114 ± 4.1	110.7 ± 4.4	0.964	0.6488	0.1943
	Thaw	104.8 ± 6.8 ^ab^	76.2 ± 8.9 ^b^	114.3 ± 8.3 ^a^	82.8 ± 5 ^b^	0.8504	0.0019	0.324
VAP, µm/s	Fresh	63.9 ± 5.9	60.4 ± 9	53.5 ± 3.2	52.6 ± 3.1	0.8117	0.6711	0.0952
	Thaw	75.4 ± 5 ^a^	49.1 ± 6.6 ^b^	78.5 ± 3.8 ^a^	56.4 ± 2.9 ^b^	0.652	0.0001	0.2794
VSL, µm/s	Fresh	40 ± 3.9 ^a^	35.5 ± 0.5 ^ab^	34 ± 3.7 ^ab^	23.7 ± 2.3 ^b^	0.4507	0.0686	0.0326
	Thaw	63.4 ± 4.3 ^a^	40 ± 8 ^b^	64.2 ± 1.9 ^a^	47.5 ± 2.4 ^b^	0.3921	0.0001	0.2924
STR, %	Fresh	66 ± 1.6	63.8 ± 7.8	70.8 ± 2.1	65 ± 2.8	0.5724	0.2152	0.3482
	Thaw	85.3 ± 2.9	67.9 ± 19.1	81.9 ± 2.6	88.5 ± 0.9	0.0184	0.2505	0.0753
LIN, %	Fresh	34.3 ± 0.8	32.2 ± 2.8	32.5 ± 2.6	27.3 ± 2.5	0.5672	0.1871	0.2141
	Thaw	63.8 ± 2.9	44.1 ± 14.6	59. ± 35	63.3 ± 1.7	0.013	0.0896	0.1092
WOB, %	Fresh	50.9 ± 1.2	49.8 ± 1.5	46.9 ± 1.8	47.4 ± 1.6	0.6511	0.8723	0.1007
	Thaw	72.1 ± 1.9	64.3 ± 1.2	69.2 ± 2.1	68.4 ± 1.2	0.0978	0.0454	0.7642

HF-GF: high-fertility and good freezability, HF-BF: high-fertility and poor freezability, LF-GF: low-fertility and good freezability, and LF-BF: low-fertility and poor freezability. Total sperm motility (MT, %), progressive motility (PM, %), curvilinear velocity (VCL, µm/s), straight-line velocity (VSL, µm/s), average path velocity (VAP, µm/s), percentage of linearity (LIN, %), percentage of straightness (STR, %), and wobble coefficient (WOB, %). Significant differences (*p* < 0.05) between groups at fresh or thaw samples are indicated with a superscript lowercase letter (a, b). Data are depicted as means ± SEM.

**Table 6:** Fluorescence microscopic in Fresh-Doses AI and thawing samples in boar classified by fertility and freezability.

		HF-GF	HF-BF	LF-GF	LF-BF	*p*-Value Interaction	*p*-Value Freezability	*p*-Value Fertility
Live. non-reacted Acr. %	Fresh	77.9 ± 2.8	82.1 ± 2.4	76.6 ± 3.9	69 ± 3.6	0.1657	0.6829	0.0996
	Thaw	56.1 ± 5.4 ^a^	9.3 ± 7.3 ^b^	61.8 ± 3.3 ^a^	49.2 ± 3.5 ^a^	0.0037	<0.0001	0.0004
Live. reacted Acr. %	Fresh	0.4 ± 0.4	0.0	0.0	0.0	0.4314	0.4314	0.4314
	Thaw	0.0	0.0	0.0	0.0			
Dead. non-reacted Acr. %	Fresh	11.5 ± 1.5	8.3 ± 3.2	9.8 ± 3.3	17 ± 3.1	0.137	0.5587	0.3067
	Thaw	26 ± 3	36.4 ± 0.4	27.3 ± 3	32.3 ± 3.7	0.495	0.0625	0.7087
Dead. reacted Acr. %	Fresh	10.3 ± 1.8	9.6 ± 5.6	13 ± 1.3	14 ± 0.6	0.6429	0.9304	0.0732
	Thaw	17.8 ± 2.6 ^b^	54.2 ± 7.9 ^a^	10.9 ± 2.3 ^b^	18.4 ± 1.5 ^b^	0.0002	<0.0001	<0.0001
Live. MITOact. %	Fresh	79.9 ± 0.3	80.3 ± 1.2	71.6 ± 4	67.9 ± 3.9	0.6256	0.6986	0.025
	Thaw	50.4 ± 5 ^a^	9.9 ± 8.1 ^b^	49 ± 2.6 ^a^	43.4 ± 2.8 ^a^	0.0023	0.0002	0.0041
Live. MITOdeact. %	Fresh	0.07 ± 0.07	0.0	0.6 ± 0.3	1.1 ± 0.6	0.607	0.702	0.1831
	Thaw	0.0 ^b^	1.8 ± 1.8 ^a^	0.0 ^b^	0.1 ± 0.1 ^ab^	0.0415	0.0253	0.0415
Dead. MITOact. %	Fresh	1 ± 0.8	1.2 ± 0.7	4.6 ± 3.8	3.5 ± 1.1	0.8076	0.8714	0.3015
	Thaw	0.3 ± 0.2	0.0	0.0	0.1 ± 0.1	0.2307	0.6234	0.6234
Dead. MITOdeact. %	Fresh	19 ± 0.7	18.4 ± 2	23.2 ± 3.3	27.5 ± 4.8	0.5968	0.6858	0.1586
	Thaw	49.4 ± 5 ^b^	88.2 ± 8.1 ^a^	51 ± 2.6 ^b^	56.4 ± 2.9 ^b^	0.0023	0.0002	0.0045
High membrane potential. %	Fresh	49.8 ± 7.1	84 ± 4.8	57.3 ± 6.8	76.6 ± 7.2	0.3762	0.0058	0.9973
	Thaw	11.8 ± 5	0.0	7.4 ± 7.1	29.1 ± 11.1	0.1139	0.6242	0.2341
Live. non-Apoptotic. %	Fresh	76.7 ± 1.8	79.1 ± 1.2	70.9 ± 3.9	68.4 ± 4.1	0.5542	0.9898	0.0628
	Thaw	50.1 ± 5.1 ^a^	11.7 ± 8.1 ^b^	48.3 ± 2.5 ^a^	43.4 ± 2.8 ^a^	0.0023	0.0003	0.0049
Live. Apoptotic. %	Fresh	3.8 ± 1.6	1.2 ± 0.1	2 ± 1.1	0.6 ± 0.3	0.6225	0.123	0.3564
	Thaw	0.3 ± 0.3	0.0	0.7 ± 0.5	0.08 ± 0.08	0.5996	0.2378	0.4647
Dead. non-Apoptotic. %	Fresh	0.0	0.0	0.1 ± 0.1	0.08 ± 0.08	0.7949	0.7949	0.3141
	Thaw	0.0	0.0	0.64 ± 0.5	0.0	0.3649	0.3649	0.3649
Dead. Apoptotic. %	Fresh	19.5 ± 0.6	19.6 ± 1.2	26.9 ± 3.9	30.9 ± 4	0.631	0.5979	0.0312
	Thaw	49.6 ± 5 ^b^	88.2 ± 8.1 ^a^	50.3 ± 2.9 ^b^	56.5 ± 2.8 ^b^	0.0034	0.0003	0.0045
Non-apoptotic. non-oxidized. %	Fresh	80.7 ± 4	81 ± 0.5	74.9 ± 5	62 ± 6.1	0.2974	0.3192	0.061
	Thaw	50.3 ± 4 ^ab^	9.4 ± 6.1^c^	57 ± 2.6 ^a^	43.1 ± 3.4 ^bc^	0.0051	<0.0001	0.0002
Non-apoptotic. oxidized. %	Fresh	0.2 ± 0.2	0.4 ± 0.4	0.6 ± 0.6	3.6 ± 2.7	0.5133	0.4498	0.3966
	Thaw	1.6 ± 0.7	0.0	0.9 ± 0.5	2 ± 0.7	0.0973	0.7472	0.4128
Apoptotic. non-oxidized. %	Fresh	0.6 ± 0.4	0.0	3.2 ± 3.2	0.7 ± 0.5	0.6483	0.4517	0.4339
	Thaw	0.0	1 ± 1	0.5 ± 0.3	0.1 ± 0.1	0.0377	0.3201	0.4964
Apoptotic. oxidized. %	Fresh	18.5 ± 4.2	18.6 ± 0.9	21.3 ± 5.4	33.7 ± 5.9	0.3346	0.3286	0.1692
	Thaw	48.1 ± 3.8 ^b^	89.5 ± 7.1 ^a^	41.7 ± 2.7 ^b^	54.8 ± 3.1 ^b^	0.0033	<0.0001	0.0001

HF-GF: high-fertility and good freezability, HF-BF: high-fertility and poor freezability, LF-GF: low-fertility and good freezability, and LF-BF: low-fertility and poor freezability. Live, non-reacted Acr (%): spermatozoa with undamaged membrane and intact acrosome; Live, reacted Acr (%): undamaged membrane and reacted acrosome; Dead, non-reacted Acr (%): damaged membrane and intact acrosome; Dead, reacted Acr (%): damaged membrane and reacted acrosome; Live, MITOact (%): live spermatozoa with active mitochondria; Live, MITOdeact (%): live spermatozoa with inactive mitochondria; Dead, MITOact (%): dead spermatozoa with active mitochondria; Dead, MITOdeact (%): dead spermatozoa with inactive mitochondria; high membrane potential: percentage of sperm displaying high membrane potential (JC-1 orange); Live, non-Apoptotic (%): live non-apoptotic spermatozoa; Live, Apoptotic (%): live apoptotic spermatozoa; Dead, non-Apoptotic (%): dead non-apoptotic spermatozoa; Dead, Apoptotic (%): dead apoptotic spermatozoa; Non-Apoptotic, Non-Oxi (%): non-apoptotic and non-oxidized spermatozoa; Non-Apoptotic, Oxi (%): non-apoptotic and oxidized spermatozoa; Apoptotic, Non-Oxi (%): apoptotic and non-oxidized spermatozoa; and Apoptotic, Oxi (%): apoptotic and oxidized spermatozoa. Significant differences (*p* < 0.05) between groups at fresh or thaw samples are indicated with a superscript lowercase letter (a, b, c). Data are depicted as means ± SEM.

## Data Availability

The original contributions presented in this study are included in the article. Further inquiries can be directed to the corresponding author(s).
